# *Salmonella typhimurium* targeting with monoclonal antibodies prevents infection in mice

**DOI:** 10.1371/journal.pntd.0011579

**Published:** 2023-12-04

**Authors:** Jie Li, Yang Yang, Zhongyi Fan, Zhiqiang Huang, Jun Chen, Qing Liu

**Affiliations:** 1 Second Affiliated Hospital, Southern University of Science and Technology, Shenzhen Third People’s Hospital, Shenzhen, China; 2 School of Medical Instrument and Food Engineering, University of Shanghai for Science and Technology, Shanghai, PR China; The University of Kansas, UNITED STATES

## Abstract

*Salmonella* is a prevalent foodborne and waterborne pathogens threating global public health and food safety. Given the diversity of Salmonella serotypes and the emergence of antibiotic-resistant strains, there is an urgent need for the development of broadly protective therapies. This study aims to prepare monoclonal antibodies (Mabs) with broad reactivity against multi-serotype *Salmonella* strains, potentially offering cross-protection. We prepared two Mabs F1D4 and B7D4 against protein FliK and BcsZ, two potential vaccine candidates against multi-serotype *Salmonella*. The two Mabs belonging to IgG1 isotype exhibited high titers of 1:256,000 and 1:512,000 respectively, as well as broad cross-reactivity against 28 different serotypes of *Salmonella* strains with percentages of 89.29% and 92.86%, correspondingly. Neutralizing effects of the two Mabs on *Salmonella* growth, adhesion, invasion and motility was evaluated in vitro using bacteriostatic and bactericidal activity with and without complement and bacterial invasion inhibition assay. Additionally, cytotoxicity assays, animal toxicity analyses, and pharmacokinetic evaluations demonstrated the safety and sustained effectiveness of both Mabs. Furthermore, F1D4 or B7D4-therapy in mice challenged with *S*. Typhimurium LT2 exhibited milder organs damage and lower *Salmonella* colonization, as well as the higher relative survival of 86.67% and 93.33% respectively. This study produced two broadly reactive and potential cross protective Mabs F1D4 and B7D4, which offered new possibilities for immunotherapy of salmonellosis.

## Author summary

Two Mabs F1D4 and B7D4 targeting the broad spectrum of vaccine proteins FliK and BcsZ separately were prepared. The two Mabs showed the potential to be broad-spectrum therapeutic antibodies for the treatment of Salmonella infections, which applied equally to several other common foodborne pathogens.

Results suggest that passive immunotherapy with F1D4 or B7D4 in S. Typhimurium LT2 infected mice can reduce the level of infection-related mortality, and both Mabs conferred protection in a dose-dependent manner.

## 1. Introduction

*Salmonella*, a genus of gram-negative, facultative anaerobic, rod-shaped bacteria that can cause illness in humans [1]. Most people infected with *Salmonella* show diarrhea, fever, and stomach cramps, sometimes even secondarily infected in urine, blood, bones, joints, or nervous system (spinal fluid and brain) [2,3]. It has been estimated that 190,000 deaths are caused by *Salmonella enterica* serovars Typhi and Paratyphi A, B and C annually, and approximately 153 million human infections and 155,000 patients deaths are caused by non-typhoidal *Salmonella* [4–6]. More than 2,600 serovars have been identified based on the recognition of specific antigens by antibodies, posing a persistent challenge for *Salmonella* detection and salmonellosis treatment [7]. Traditionally, antibiotic drugs used to be the optimal and most cost-effective to control *Salmonella* infections [8]. However, widespread antibiotic resistance threatens the continued efficacy of antimicrobial therapy to *Salmonella* [9,10]. Antimicrobial resistance to several classes of antibiotics such as penicillins, tetracyclines, fluoroquinolones, sulfonamides, aminoglycosides, and cephalosporins is another major concern in treatment of *Salmonella* infections [11]. Ty21a, a live licensed attenuated vaccine capable of inducing expansion of T cells as well as antibodies against *Salmonella*, exhibited a 3-year efficacy against typhoid of 67% [12] In addition, WHO has prequalified two typhoid conjugate vaccines, Typbar-TCV and TYPHIBEV. These vaccines can be administered to individuals aged 6 months or older and included in routine immunization programs. A single dose is safe and effective for children, with an antibody response lasting up to 7 years. Co-administration with other vaccines is also possible [13].

With the development in preparation and reform of Mabs, which have been applied in a variety of scientific areas including immunodiagnostic procedures [14] detection of pathogenic microorganisms, especially for therapeutic purposes [15–17]. According to “Antibodies to Watch”, as of 2023, over 1200 antibody therapeutics currently in clinical studies and ~175 that are in regulatory review or approved [18]. Passive immunization has been successfully used as an alternate method of prophylaxis against several gastrointestinal pathogens such as *Campylobacter*, *E*. *coli*, *rotavirus*, and *coronavirus* [17]. The potential of using MAbs as prophylactic or therapeutic treatment for salmonellosis is promising, given their lack of susceptibility to bacterial resistance and toxicity hurdles of small molecules [19]. Nowadays, the development of therapeutic antibodies for treating bacterial infections remains in its infancy. Effective wide-spectrum Mabs targeting multiple serovars of *Salmonella* are still being prepared. Sierocki et.al [20] produced an antibody targeting type III secretion system induces broad protection against *Salmonella* and *Shigella* infections, which provides the first in vivo experimental evidence of the importance of this common region in the mechanism of virulence of *Salmonella* and Shigella and opens the way to the development of cross-protective therapeutic agents. However, the relatively low identity sequence (approximately 40%) of the immunogen SipD may not be sufficient to address the complexity of *Salmonella* serotypes. Reddy et.al [21] studied the functional characterization of a broad and potent neutralizing monoclonal antibody directed against outer membrane protein (OMP) of *Salmonella* typhimurium. The development of broadly reactive and cross protective Sal-06mAb opens new possibilities for immunotherapy of sepsis caused by Gram-negative Enterobacteriaceae members.

In the present study, we utilized FliK and BcsZ as vaccine candidates for *Salmonella*, which were identified through *in silico* computational modeling and experimental work in a previous study [22]. Two Mabs, namely, F1D4 and B7D4, were generated with broad reactivity against multi-serotype *Salmonella* strains and potential cross-protection. The anti-*Salmonella* properties of the two mAbs were examined through cross-reactivity, antibacterial activity, invasion inhibition assays, and validated by in vivo experiments.

## 2. Materials and methods

### 2.1. Bacterial and cell lines

All *Salmonella* strains (Table 1) utilized in this study were obtained from the strain bank of the University of Shanghai for Science and Technology. A total of 28 strains of *Salmonella* were cultured overnight in LB medium at 37°C. All cells used in this research, including SP2/0 myeloma tumor cells, hybridoma cells, Caco2 and RAW264.7 cells, were cultured in DMEM supplemented with 10% fetal bovine serum at 37°C under a humidified atmosphere containing 5% CO^2^.

**Table 1:** *Salmonella* strains used in this study.

NO	Strains	Strain ID	NO	Strains	Strain ID
1	*Salmonella* Choleraesuis	CICC 21493	15	*Salmonella* Paratyphi C	IQCC 10527
2	*Salmonella* Typhimurium	ATCC 14028	16	*Salmonella* Typhi	CMCC50071
3	*Salmonella* Typhimurium	CMCC(B) 50115	17	*Salmonella* Enteritidis	ATCC 13076
4	*Salmonella* Typhimurium	IQCC 10503	18	*Salmonella* Enteritidis	IQCC 10512
5	*Salmonella* Typhimurium	CMCC 50098	19	*Salmonella* Enteritidis	IQCC 10528
6	*Salmonella* Typhimurium	LT2	20	*Salmonella* Bovismorbificans	IQCC 10510
7	*Salmonella* Paratyphi A	IQCC 10508	21	*Salmonella* Thompson	IQCC 10514
8	*Salmonella* Paratyphi A	IQCC 30537	22	*Salmonella* Dublin	IQCC 10523
9	*Salmonella* Paratyphi A	IQCC 30538	23	*Salmonella* Saintpaul	IQCC 10529
10	*Salmonella* Paratyphi A	CMCC(B) 50093	24	*Salmonella* Kentucky	IQCC 10530
11	*Salmonella* Paratyphi B	CMCC(B) 50094	25	*Salmonella* Heidelberg	IQCC 10531
12	*Salmonella* Paratyphi B	CICC 21495	26	*Salmonella* Blockley	IQCC 10532
13	*Salmonella* Paratyphi B	IQCC 10504	27	*Salmonella* Aberdeen	IQCC 10511
14	*Salmonella* Paratyphi C	CICC 21512	28	*Salmonella* Anatum	IQCC 10509

### 2.2. Animal studies and ethics statement

All experiments utilizing mice were conducted in strict accordance with the regulations of the “Institutional Animal Care and Use Committee at Tongji University School of Medicine” with the permitted number: TJLAC-018-025. A total of 300 female BALB/c mice (6–8 weeks old, weighing 18±1 g) were purchased from Jie Si Jie Laboratory Animal Ltd. (Shanghai, China). all mice in each group were euthanized in a CO2 chamber.

### 2.3. Antigen preparation

In a previous study, FliK and BcsZ were identified as broad-spectrum vaccine candidates of *Salmonella*. The sequences from Uniprot are listed in S1 Table. In this study, E. coli BL21 with pET30a vector was utilized to express FliK and BcsZ, which were subsequently purified using Ni-NTA affinity chromatography columns (Sangon, Shanghai), following previously established protocols.

### 2.4. Production and characterization of Mabs

Mabs were generated through hybridoma technology. Female BALB/c mice, aged six weeks, were immunized with FliK or BcsZ intramuscularly in the hind leg (20 μg of recombinant protein in 50 μL of QuickAntibody-Mouse 3W adjuvant [Biodragon Immunotechnologies Ltd., Beijing, China]) at two-week intervals. Three days after an additional booster immunization, fusion of splenocytes with SP2/0 myeloma cells was conducted. Positive monoclonal hybridoma lines were screened by HAT/HT and ELISA. Mabs were generated by injecting Hybridoma cell clones into mice intraperitoneally to produce ascites, which were then purified using a protein-G column (GE). Purity and molecular weight were assessed via SDS-PAGE analysis. The Mabs subtype was determined using a mouse monoclonal antibody isotyping kit (Bio-Rad, America) in accordance with the manufacturer’s instructions."

### 2.5. ELISA

96-well plates (Costar, USA) were coated with purified proteins (30 ng/well) or bacteria (10^8^ CFU/well) and incubated overnight at 4°C. Subsequently, individual hybridoma culture supernatants or purified monoclonal antibodies were added to each well (100 μl/well), followed by incubation with horseradish peroxidase-conjugated goat anti-mouse IgG (1:10,000) (Sigma, USA). The negative control consisted of serum from mice injected with PBS. The substrate reaction’s optical density (OD) was measured at 450 nm using a multifunctional microplate reader (SpectraMax/n2, USA). Samples were deemed positive if the OD_450 nm_ of the lowest dilution was 2.1 times higher than that of negative control wells. The relative OD_450 nm_ value was calculated by subtracting 2.1 times the OD_450 nm_ value of serum from mock-immunized mice with PBS from the OD_450 nm_ value of Mabs [23].


$$\text{Relative}\;{\text{OD}}_{450\;\text{nm}}={\text{OD}}_{450\;\text{nm}}\;\text{of}\;\text{Mabs}–2.1\times {\text{OD}}_{450\;\text{nm}}\;\text{of}\;\text{PBS}$$

### 2.6 Invasion, adhesion, and intracellular killing assay

The effect of the two Mabs on bacterial invasion was determined using the gentamicin protection assay, as described previously, with some modifcations. Briefy, F1D4 or B7D4 (200 μg/mL, 150 μg/mL, 100 μg/mL, 50 μg/mL) was added to fully confuent Caco-2 cells (10^5^ cells/mL) and incubated for 1 h separately. *S*. Typhimurium LT2 (10^7^ CFU/mL) were added and incubated for another 1.5 h after being centrifuged at 500 × g for 5 min. Gentamicin (100 μg/mL) was added and incubated for 30 min before cells were lysed with 0.1% Triton x-100 for 20 min. Finally, the suspension was serially diluted and cultured on LB agar plates for viable counting of bacteria after overnight incubation.

The same procedure was followed for the adhesion inhibition assay, except for treatment with gentamicin. The experiments were performed three times in duplicate. Similarly, for the intracellular killing experiment, RAW 264.7 cells (10^5^ cells/mL) were cultured in 24-well plates. The cells were treated with *S*. Typhimurium LT2 (10^7^ CFU/mL) and incubated for 1 h after centrifugation at 500 g for 5 min. After the cells were washed, F1D4 or B7D4 (200 μg/mL, 150 μg/mL, 100 μg/mL, 50 μg/mL) was added and incubated for 1 h at 37°C and 5% CO2. Finally, cells were treated with gentamicin (100 μg/mL) for 1 h and lysed with 0.1% of Triton×100 before being serially diluted and plated on LB agar. For all experiments, cells infected with *S*. Typhimurium LT2 without treatment and those treated with sub-MIC of MRB were used as the control.

### 2.7 Confocal microscopy

The protective ability of F1D4 and B7D4 against *Salmonella* invasion was observed by confocal microscopy. The experimental method was performed following the reference [24]. The murine macrophage cell line RAW264.7 (10^5^ cells/mL) were seeded and incubated in a confocal dish overnight at 37°C, 5% CO^2^. F1D4 or B7D4 (at concentrations of 200 μg/mL, 150 μg/mL, 100 μg/mL, and 50 μg/mL) were added and incubated for four hours. S. Typhimurium LT2 labeled with FITC-D-Lys (final concentration: 0.1 mM) was then introduced to the confocal dish and allowed to incubate at 37°C for one-and-a-half hours. followed by fixation of the cells in 4% paraformaldehyde for 15 min and washing with PBS three times before visualization under a laser scanning confocal microscope.

### 2.8 Motility assay

As bacterial motility plays a crucial role in its virulence, we aimed to investigate the impact of antibodies on *Salmonella*’s motility. The motility assay was performed using LB medium containing 0.4% (W/V) agar on 6-well plate (9.6 cm^2^ per well). Briefly, an 8-hour culture of S. Typhimurium LT2 was inoculated with F1B4 or B7D4 at concentrations of 100 μg/mL, 75 μg/mL, 50 μg/mL or 25 μg/mL respectively, while medium without antibody served as the control. The plates were then incubated overnight at 37°C, and the diameter of bacterial spread was measured using calipers.

### 2.9 Cytotoxicity assay, pathomorphology analysis, and pharmacokinetic evaluation of the two Mabs

The cytotoxicity of Mabs drugs was assessed in vitro using a CCK8 assay. Caco2 cells were seeded at a density of 10^5^ cells/mL in a 96-well plate and incubated overnight. The following day, fresh medium containing varying concentrations (200 μg/mL, 150 μg/mL, 100 μg/mL or 50 μg/mL) of the Mabs was added and incubated overnight. Subsequently, each well received fresh medium with the addition of 10 μL CCK8 reagent (MedChemExpress(MCE), USA) and was then incubated for one hour before measuring optical density at 450 nm.

Measurement of the serum concentrations of administered antibodies is a general tool to evaluate their persistence in circulation. To monitor the serum clearance of Mabs, 500 μg of purified F1D4 and B7D4 were administered through the intraperitoneal route. Blood samples were taken from from the same cohort of mice (four mouse each group) at the retroorbital sinus on days 0, 1, 3, 5, 7, 9, 11, 13, 15, 17 and 21. The residue of the two Mabs were determined by ELISA techniques from plasma prepared from these samples. Additionally, general condition, behavioral changes, food and water consumption, visible signs of intoxication, and animal death were monitored daily during 21 days post-injection of F1D4, B7D4 or PBS. The liver, spleen, and cecum of mice were subjected to pathological analysis on the 7th day following intraperitoneal injection of antibodies in order to determine whether antibody-mediated immunity had any adverse effects on these organs. liver, spleen and caecum were isolated to hematoxylin and eosin (H&E) staining. Histopathological changes were examined by fluorescence microscopy (Leica DM2500), and representative images were selected for analysis.

### 2.10. Passive immune protection

The 50% lethal dose (LD50) of *S*. Typhimurium LT2 for BALB/c mice was determined to be 6.4×10^4^ CFU using Karber’s method in previous studies (Li et al. 2021)[22]. To assess the efficacy of Mabs in providing passive immune protection, BALB/c mice were intraperitoneally challenged with S. Typhimurium LT2 at a dose 10 times higher than the lethal dose in 100 μL of PBS. After one hour, mice were administered either 300 or 500 μg/mouse of the Mabs via intraperitoneal injection, while control mice received only 100 μL of PBS. The group that demonstrated superior results was subjected to another round of passive immune protection testing. Over a period of thirty days, morbidity and mortality rates were monitored daily, and the relative percent survival (RPS) was calculated according to Eq (1):

$$RPS=\frac{1-MR\left(EG\right)}{MR\left(NC\right)}$$ (1)

where *MR*(*EG*) refers to the mortality rate of the experiment group, and *MR*(*NC*) refers to the mortality rate of the negative control (NC) group.

The daily determination of the number and timing of mouse mortality over a 4-week period enabled calculation of the extension in survival time resulting from treatment with Mabs F1D4, B7D4 or PBS following *Salmonella* infection:

$$Life\;extension\;rate=\left(Vm-Cm\right)/Vm\times 100\%$$ (2)

Where Vm refer to the mean survival time in the vaccine group, and Cm refers to mean survival time in the control group.

Simultaneously, to comprehensively evaluate the therapeutic efficacy of the antibodies, aseptic collection of spleen, liver, cecum and feces from infected mice was conducted on days 5, 7, 10 and 15 post-infection. Bacterial load enumeration involved homogenization and lysis of organs with 1% Triton-X100 buffer followed by centrifugation at 300 g for five minutes to remove tissue debris. Finally, the lysates were serially diluted and plated onto CHROM agar for *Salmonella*. The resulting colonies were then enumerated after overnight incubation at 37°C. To assess the impact of infection on the spleen and liver, histopathological examinations were performed on the spleen and liver of mice treated with Mab and PBS (n = 3) 7 days after intraperitoneal challenge with 6.4×10^5^ CFU of *S*. Typhimurium LT2. The excised organs were fixed in 4% paraformaldehyde for over 24 hours before being subjected pharmacokinetic analysis.

### 2.11. Statistical analysis

All statistical analyses were conducted using GraphPad Prism 5. All ELISAs were performed in triplicate, and numerical data are presented as mean±SD. The significance of differences between the respective Mab treatment group and control group was assessed by an unpaired Student’s t-test, with ^ns^P>0.05 indicating no significant difference, *P≤0.05 indicating a significant difference **P≤0.01 indicating a highly significant difference, and ***P≤0.005 indicating an extremely significant difference.

## 3. Result

### 3.1. Preparation, purification and identification of Mabs

**Fig 1:**
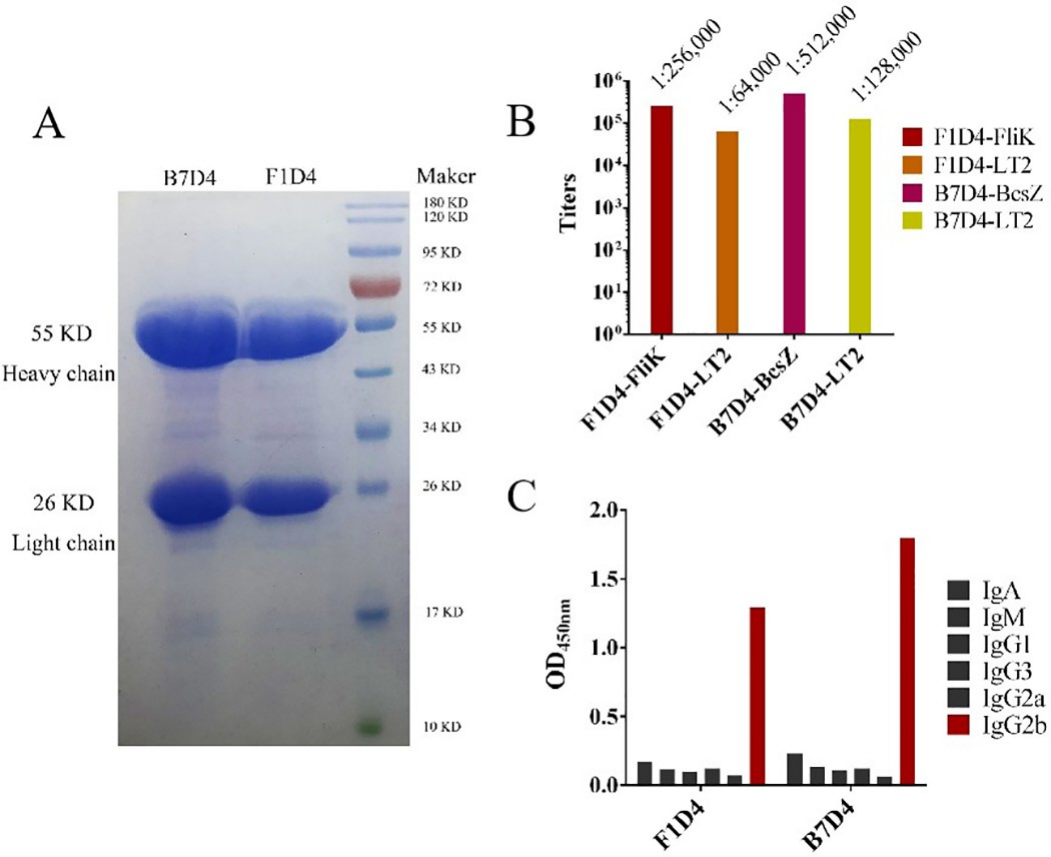
Purification and identification of monoclonal antibodies (A) displays the SDS-PAGE analysis of purified Mabs F1D4 and B7D4, with a Prestained 180 kD Protein Ladder (in KD of 26, 34, 43, 55, 72, 95, 130, and 180 from bottom to top) (Beyotime Biotechnology, Shanghai). The high purity and molecular weight of the heavy/light chains of Mabs are evident in the image. (B) ELISA analysis shows titers between Mabs and corresponding protein antigens or S. Typhimurium LT2. (C) ELISA analysis reveals Mab subtypes using HRP-labeled goat anti-mouse IgA/IgM/IgG1/IgG3/IgG.

In our study, we successfully generated two hybridoma cell lines that produce stable antibodies targeting FliK and BcsZ using hybridoma technology. The monoclonal antibodies (mAbs) obtained were designated as F1D4 and B7D4, respectively. The high purity of the mAbs’ light/heavy chains is demonstrated in Fig 1A and S1 Fig.

### 3.2. Mabs characterization

The ELISA results revealed that the titers of purified antibodies (F1D4 and B7D4, 2 mg/mL) against their corresponding immunogen proteins FliK and BcsZ were 1:256,00 and 1:512,000 respectively. In comparison to *S*. Typhimurium LT2, these values were significantly higher at 1:64,000 and 1:128,000 for FliK and BcsZ antibodies, respectively (Fig 1B). Both Mabs demonstrated high affinity towards their respective immunogen and *S*. Typhimurium LT2, indicating that the specific binding sites of the antibodies may be located on surface of the bacteria—a prerequisite for therapeutic antibody drugs. Additionally, both antibodies were identified as IgG2b subtype (Fig 1C), which is advantageous due to its ability to transfer through placenta and protect fetuses^3^.

### 3.3. Broad cross-reactivity of Mabs against *Salmonella* strains

**Fig 2:**
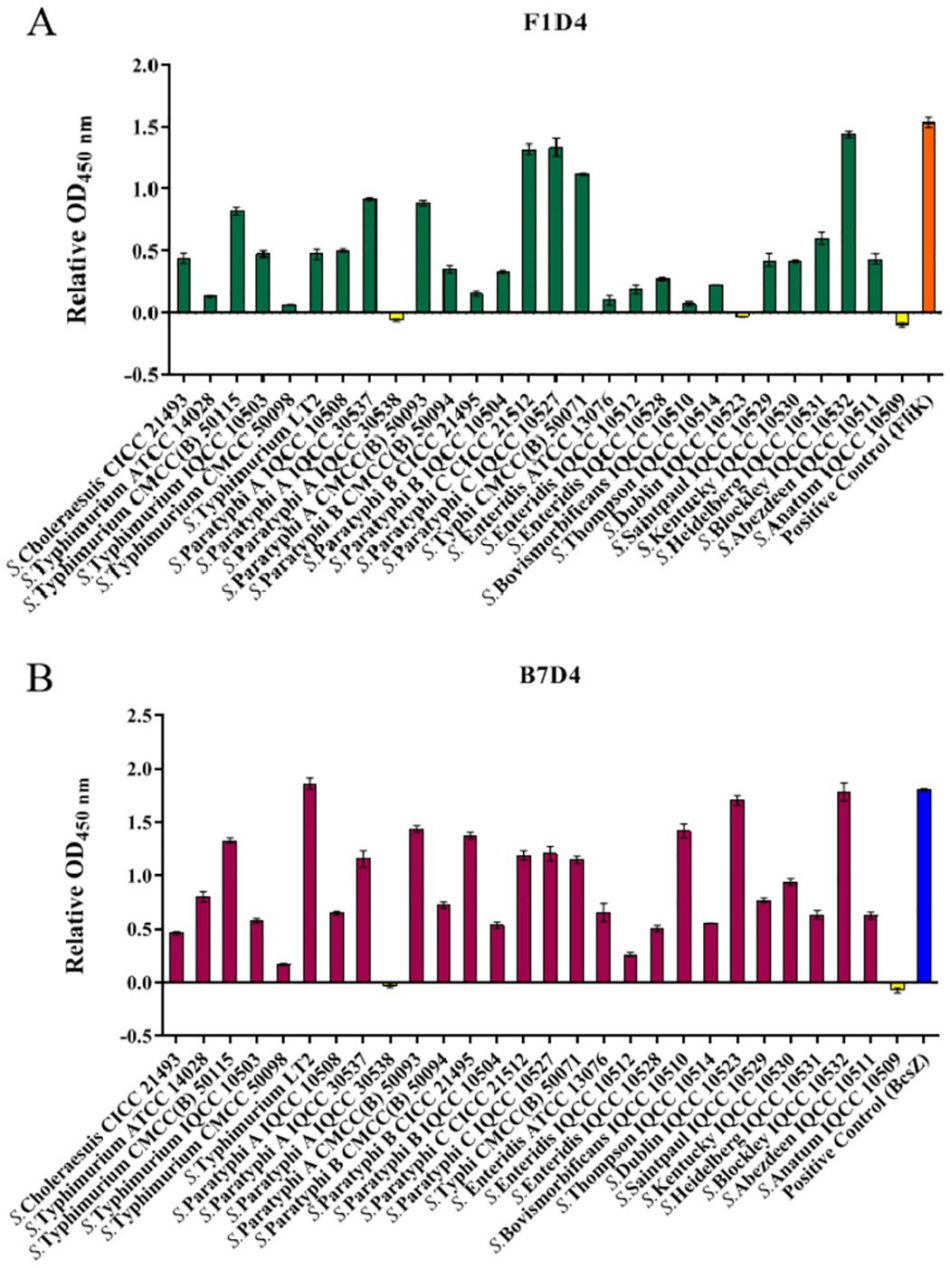
ELISA analysis was conducted to assess the cross-reactivity between Mabs F1D4 (A) and B7D4 (B) with 28 *Salmonella* strains, using corresponding protein (FliK (orange) or BcsZ (blue) as positive controls. The x-axis represents the 28 strains of *Salmonella* coated on 96-well plates and reacted with Mab at a dilution of 1:1000. Negative control (NC) involved reaction between target protein and antisera from mice mock-vaccinated with PBS. Positive control was established by reacting the target protein with the Mab. The y-axis represents the relative OD _450 nm_, which is calculated as the difference between OD_450 nm_ of tested samples and 2.1 times of NC.

The binding results of the two Mabs (F1D4 and B7D4) against 28 *Salmonella* strains (Table 1) and the corresponding proteins (FliK and BcsZ, as positive control) are presented in Fig 2. F1D4 exhibited cross-reacted with 25 out of the tested 28 strains, providing a coverage of 89.29% among *Salmonella* strains examined (Fig 2A). On the other hand, B7D4 demonstrated high cross-reacted with all but one of the tested *Salmonella* strains, resulting in an impressive cross-reactivity rate of 96.43% (Fig 2B). Furthermore, the cross-reactivity of the two Mabs against 15 non-*Salmonella* bacterial strains (listed in S1 Table) was analysed. The results, shown in S2 Fig demonstrate that these antibodies reproducibly cross-reacted with other bacteria strain including *Escherichia coli*, *Cronobacter sakazakii*, *Vibrio parahaemolyticus* and *Shigella flexneri*. These findings suggest that the Mabs possess the ability to recognize shared epitopes on the surface of diverse *Salmonella* serotypes and other non-*Salmonella* strains, indicating their potential as broad-spectrum therapeutic agents for treating *Salmonella* infections as well as several other common foodborne pathogens.

### 3.4 Mabs exhibit inhibitory effects on the motility, adhesion and invasion of *S*. Typhimurium LT2 in vitro

**Fig 3:**
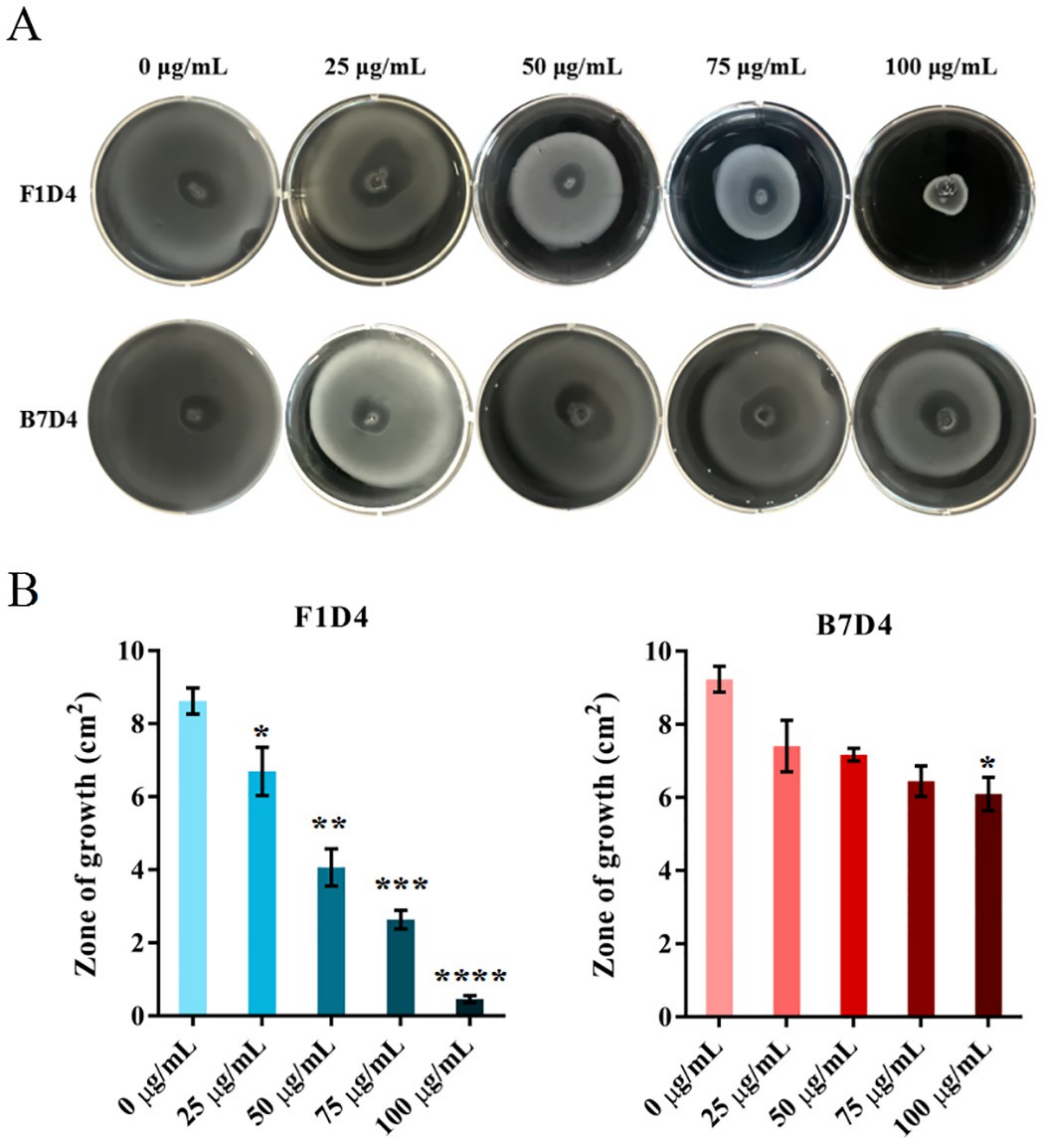
Inhibition of *S*. Typhimurium LT2 motility by F1D4 or B7D4 with a series of concentrations. (A) Inhibition of *S*. Typhimurium LT2 motility on semi-solid LB plate, *S*. Typhimurium LT2 (10^7^ CFU/mL) were inoculated at the center of the LB plate containing 0.4% agar and incubated overnight at 37°C.

F1D4 was found to inhibit the motility of *S*. Typhimurium LT2, as demonstrated in Fig 3A and 3B. Furthermore, treatment with higher concentrations of the antibody resulted in a corresponding decrease in the growth zone of *S*. Typhimurium LT2. In contrast, untreated *Salmonella* exhibited full growth on 9.6 cm^2^ petri dishes. These findings suggest that antibody F1D4 targets a critical site on the flagellate protein FLIK, which plays an essential role in *Salmonella* motility. Unfortunately, B7D4 exhibited no inhibitory effect on the motility of *Salmonella*.

**Fig 4:**
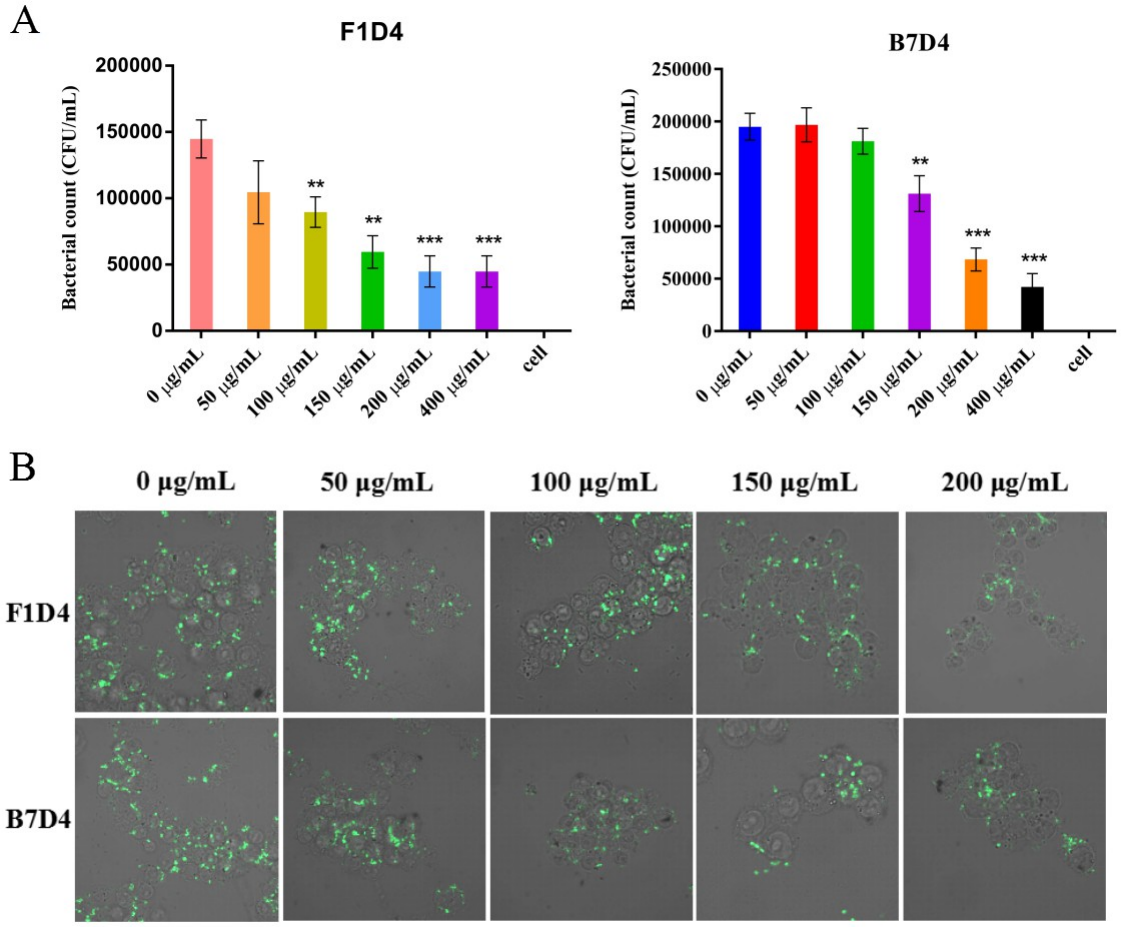
Inhibitory effects of antibodies on adhesion and invasion of *Salmonella Typhimurium*. (A). Effect of F1D4 or B7D4 on the invasion of *S*. Typhimurium LT2 with 10^7^ CFU/mL on Caco-2 cells; B. Confocal microscopy of Raw 264.7 cells infected with with 10^7^ CFU/mL of *S*. Typhimurium LT2. (Green, bacteria; image: ×1000 magnifcation; representative images of three different experiments are presented).

Inhibition of bacterial adhesion and invasion was assessed using a gentamicin protection assay. F1D4 and B7D4 both showed extremely significant inhibitory activity against adhesion of *S*. Typhimurium LT2 at concentration of 200 and 400 μg/mL(Fig 4A and 4B). The inhibition of adhesion by the Mabs treatment in a dose-dependent manner. Adhesion and invasion of *Salmonella* to the host cells was confrmed via confocal microscopy shown in Fig 4B. The bacteria were stained with FITC-D-Lys. Concurrently, confocal microscopy revealed a reduction in the number of live invading bacteria after treatment with both F1D4 and B7D4.

### 3.5 Cytotoxicity assay, pathomorphology analysis, and pharmacokinetic evaluation of the two Mabs

**Fig 5:**
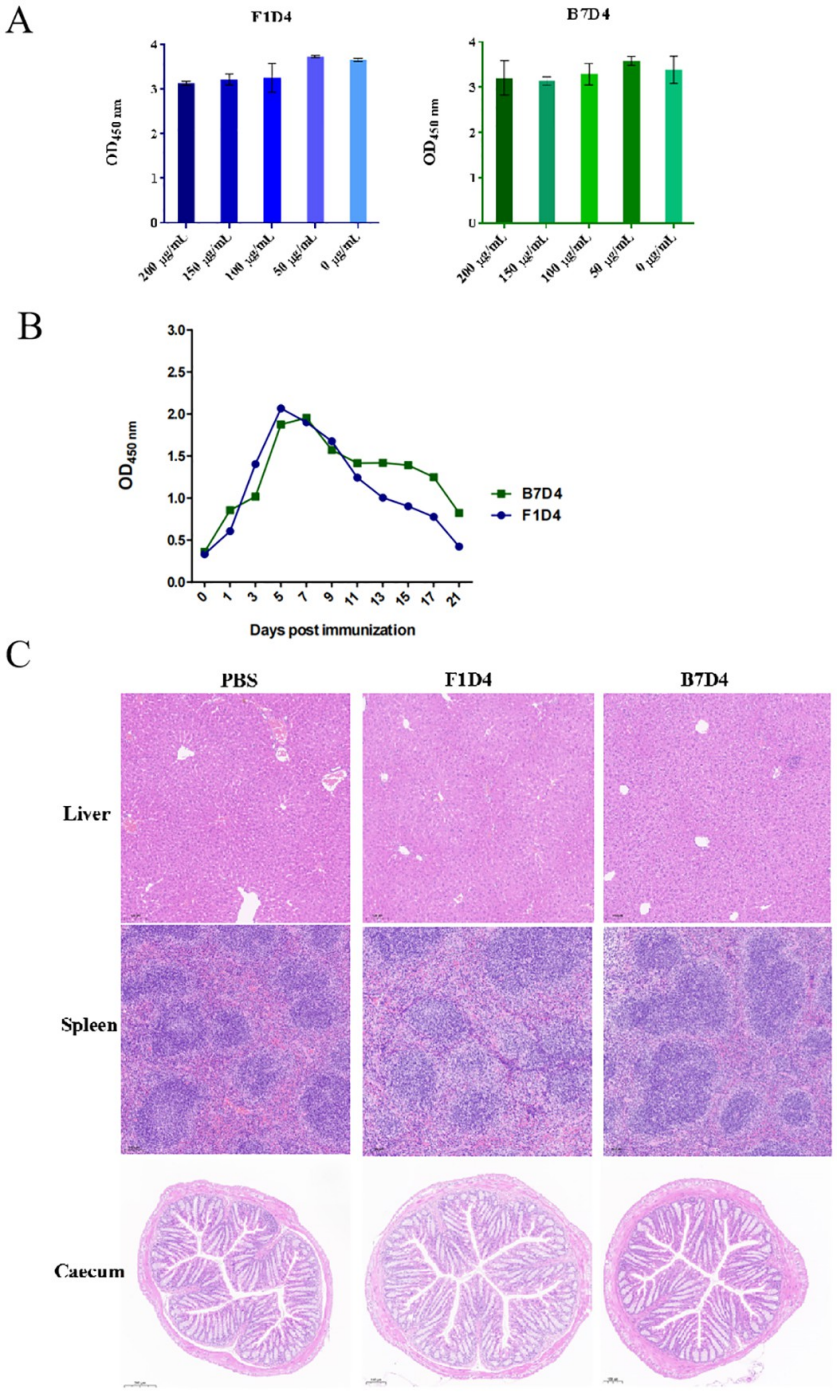
Safety and pharmacokinetic analysis of immunotherapeutic Mabs (A) Survival of Caco2 cell after co-culturing with a series of different concentrations antibodies. (B) Antibody titers-time profiles in the bloodstream. Blood samples were taken from the retroorbital sinus at the time points indicated by the symbols. The titers of Mabs were determined by ELISA techniques, as described in Subheading 2.9. (C) Representative histopathology images of liver, spleen, and cecum sections from mice immunized with Mabs are presented in H&E staining. The scale bar is set at 100 μm for liver and spleen, and 200 μm for cecum.

The cytotoxicity of F1D4 and B7D4 on mammalian cells was assessed using Caco2 cells at a series concentrations, the survival rate was measured by OD450nm. Neither Mab exhibited significant toxicity even at a higher concentration (200 μg/mL) as shown in Fig 5A. Furthermore, representative pathological images of liver, spleen, and cecum from mice immunized with Mabs showed no discernible damage compared to those immunized with PBS (Fig 5C). The immunized mice exhibited no abnormal conditions, such as decreased appetite, rough hair or diarrhea during the 21-day observation period.

In Fig 5B we show results of antibody titers-time profiles in the bloodstream, in which 500 μg of F1D4 and B7D4 were immunized intraperitoneally. We monitored the Mabs titers on days 0, 1, 3, 5, 7, 9, 11, 13, 15, 17 and 21 post-immunization. From these data shown in Fig 5B, it is apparent that the serum antibody level peaked at day 7–8 and subsequently declined over the following days, with F1D4 reaching a nadir on day 21 while B7D4 maintained an OD _450nm_ of approximately.

### 3.6. Protective effect of the Mabs in vivo

To evaluate the protective effects of Mabs F1D4 and B7D4 in vivo, We evaluated their efficacy of Mab F1D4 and B7D4 against *S*. Typhimurium LT2 infection in mice by infecting them with 6.4×105 CFU of S. Typhimurium LT2, then treating them intraperitoneally with either Mab F1D4 or B7D4 at a dose of 300 or 500 μg. The general behavior and weight of the mice were monitored daily after challenge and treatment, while the number of dead mice over a period of four weeks was recorded to determine efficacy.

**Fig 6:**
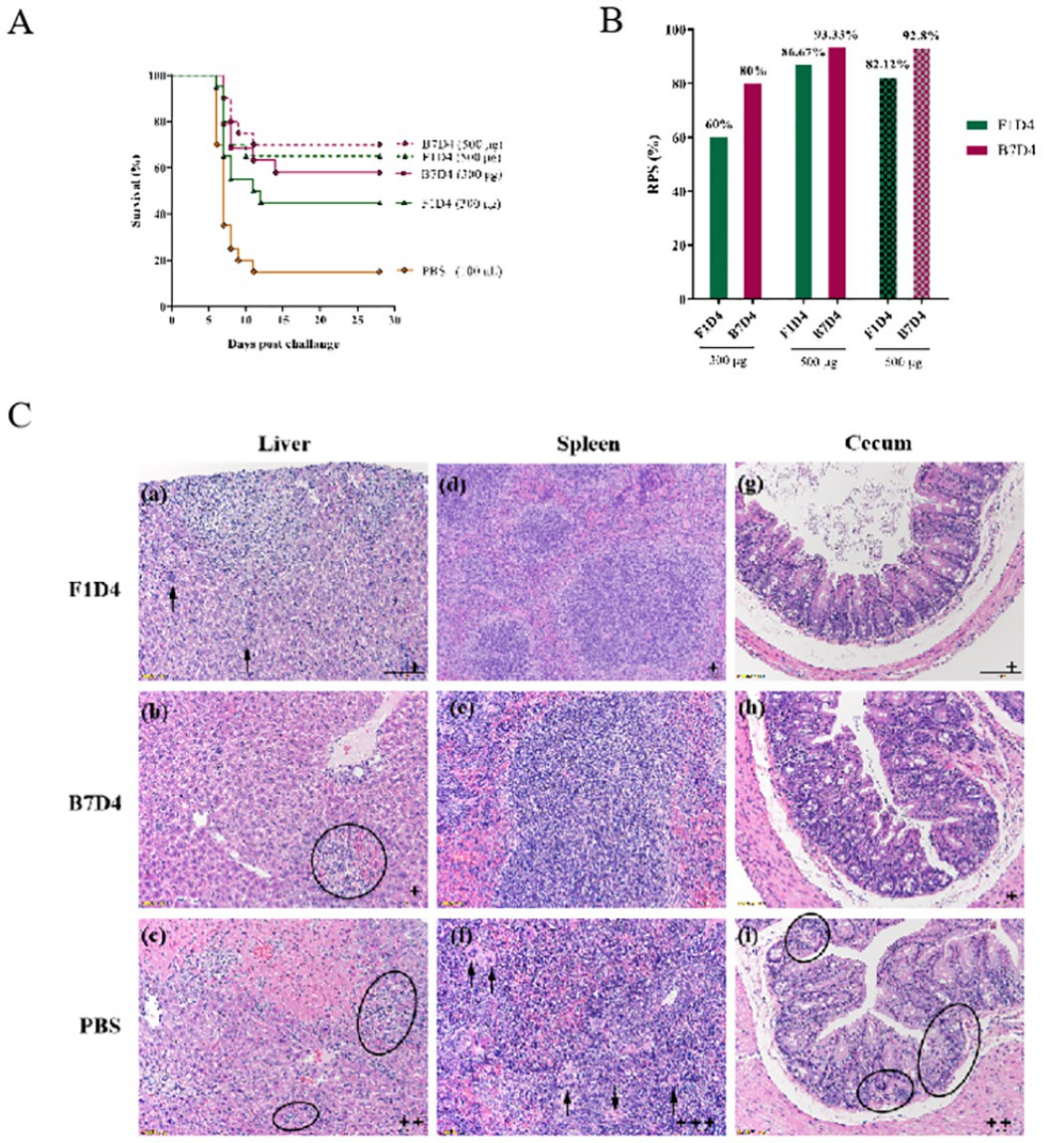
Protection against *S*. Typhimurium LT2 in mice by Mabs. Survival (A) and relative percent survival (RPS) (B) Survival analyses of mice (n = 20 per group) infected intraperitoneally with 6.4×10^5^ CFU of *S*. Typhimurium LT2, after 1 h, mice were injected intraperitoneally with either 300 or 500 μg/mouse of Mabs F1D4 or B7D4, while control mice received only PBS injection. Mice were monitored daily for morbidity and mortality over a period of 30 days. (C) Representative histopathology images (H&E, 200×, scale bar = 100 μm) of liver, spleen and cecum sections from mice challenged with 6.4×10^5^ CFU of *S*. Typhimurium LT2 and treated intraperitoneal with F1D4, B7D4, or PBS on day 7 are presented in the first and second columns respectively. The liver sections exhibit focal necrotic areas accompanied by inflammatory cell infiltration (arrows). The spleen sections show multinucleated giant cells along with sparse lymphocytes (arrows) and the splenic medulla displays a disordered and chaotic appearance. In the cecum, there was a reduction in glandular goblet cells within the midgut mucosa, infiltration of a small number of lymphocytes in the lamina propria, and deformation (elliptical) of crypt structure. Damage was scored in the bottom right corner of each Fig, “+, ++, +++”indicate an increased severity successively.

Approximately four days post-infection, the mice challenged with *Salmonella* displayed symptoms including lethargy, reduced activity levels, decreased appetite and loose stools, as well as varying degrees of weight loss over the observation period compared to control group mice. The majority of deaths occurred between days seven and eight after which there was a gradual recovery in the surviving mice. The survival rates of mice in the Mab-treated and PBS-mock groups are presented in Fig 6A. Administration of a dose of 500 μg of either Mab F1D4 or B7D4 resulted significantly higher survival rates (65% and 70%, respectively) compared to the control group (25%). Moreover, this dosage conferred superior protection than the lower dose of 300 μg for both Mabs (45% and 60%, respectively). To demonstrate the stable therapeutic efficacy of both Mabs, a subsequent immunoprotective assay was performed utilizing a dosage of 500 μg for either Mab F1D4 or B7D4. As depicted in Fig 6B, favorable outcomes were obtained again with mean RPS values of 84.39% and 93.07%, respectively. Table 2 presents the life extension rates of *Salmonella*-infected mice treated with Mab F1D4, B7D4, or PBS. The mean extended survival time within 30 days was significantly prolonged by 8.25 and 9.5 days with a dose of 500 μg for Mabs F1D4 and B7D4, respectively. These findings suggest that passive immunotherapy using F1D4 or B7D4 after *S*. Typhimurium LT2 infection can effectively reduce infection-related mortality and increase the survival time of *Salmonella*-infected mice.

**Table 2:** The mean of the extended survival time of treated mice.

Group	Dose	Strains (Dose)	No (Start/End)	MST (d)	Life extension rate (%)
PBS	100 μL	*S*. Typhimurium LT2 (6.4×10^5^ CFU)	20/5	12.6	/
F1D4	300 μg		20/9	16.95	25.66
B7D4			20/12	18.85	33.16
F1D4	500 μg		20/13	20.85	39.57
B7D4			20/14	22.1	42.99

*#* MST, mean survival time. Life extension rate = (Vm-Cm)/Vm×100%, where Vm refer to the mean survival time in the vaccine group, and Cm refers to mean survival time in the control group.

### 3.7. Bacterial loads and histopathological analysis

**Fig 7:**
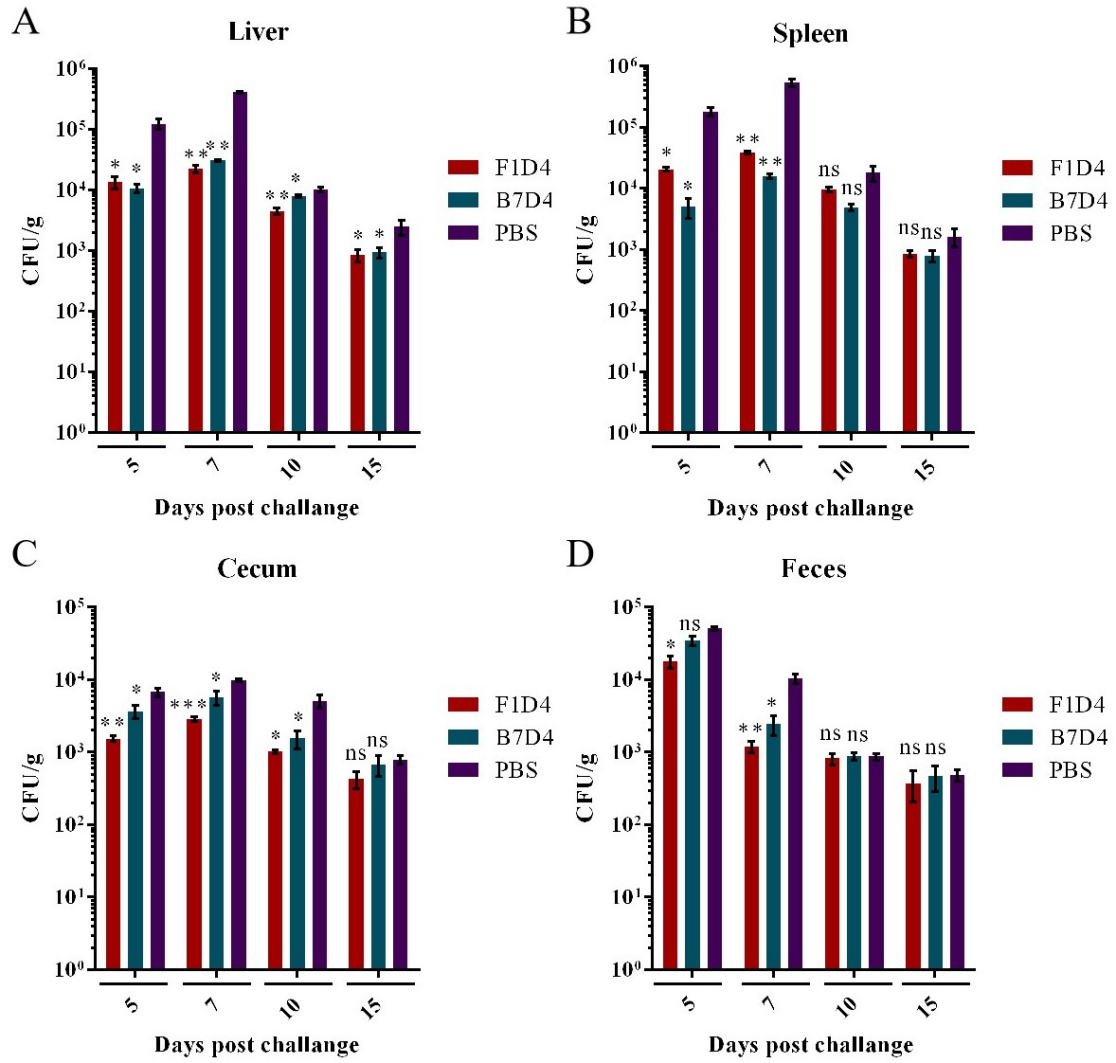
Bacterial load (CFU/g) in the liver (A), spleen (B), cecum (C), and feces (D) of mice (n = 3) treated with Mab F1D4 (300 μg) or B7D4 (300 μg) and PBS (100 μL) 1 h post challenged with *S*. Typhimurium LT2 (6.4×10^4^ CFU). The bacterial load in organs of mice was determined on days 5, 7, 10, and 15 after challenged by CHROM Agar *Salmonella*. All data are presented as mean ± SD. ^ns^ p>0.05, *p<0.05, **p<0.01, or ***p<0.005 compared with PBS group.

Infection with *S*. Typhimurium LT2 causes a systemic disease characterized by rapid bacterial multiplication in the intestinal tract, liver, spleen and cecum, resulting in hepatomegaly, splenomegaly, and the formation of acute abscesses. Encouragingly, treatment with either Mab F1D4 (300 μg) or B7D4 (300 μg) decreased the colonization of *Salmonella* in organs, thus minimized the damage caused by *S*. Typhimurium LT2 (6.4×10^5^ CFU) in treated mice compared with mice mock-treated with PBS (Fig 7). Concretely, Mabs treatment resulted in a 10^1^–10^2^ log-fold reduction of *Salmonella* CFUs per gram of spleen, liver, cecum and feces in comparison to control mice on post-treatment days 5 and 7 (Fig 7A, 7B and 7C). Surviving PBS-treated mice also gradually eliminated the invasive *Salmonella* via the immune system of mice. Additionally, numerous *Salmonella* were detected in feces on the 5th day after *Salmonella* infection when infected mice typically exhibitted severe watery diarrhea. The *Salmonella* load in the feces of the antibody-treated group was lower than that of the PBS-treated group on days 5 and 10 (Fig 7D). Indicating a significant impact of Mabs F1D4 or B7D4 on early-stage colonization of *Salmonella* in organs. However, passive antibody immunization did not enhance efflux of *Salmonella* from the intestinal pathway in mice examined in this study (Fig 7D).

Simultaneously, we evaluated organ injury and inflammatory cell infiltration by means of H&E staining. Representative microscopy slides of liver, spleen and cecum tissues (showed in Fig 6C) from mice 7 days post-infection with *S*. Typhimurium LT2 were assessed for extent of necrosis and degree of lymphocytic infiltration based on a scoring matrix in Table 3. Liver sections were examined for inflammation and focal necrotic areas, spleen sections were evaluated for lymphoid necrosis and the number of mononuclear macrophages, and cecum sections underwent examination for lymphocyte infiltration and changes in crypt structure, all the corresponding scoring values were also registered in Table 4. Mice treated with F1D4 or B7D4 exhibited milder liver injury, characterized with sparse inflammatory infiltrate and occasional necrotic foci (+). In contrast, the group treated with PBS exhibited evidence of severe liver damage, as indicated by multiple foci of hepatocyte necrosis as well as obvious inflammatory infiltration (++). The splenic architecture of mice in the control group was disorganized histologically, with an indistinct demarcation between the cortex and medulla, enlarged red pulp and disorganized medullary region. Additionally, there was significant lymphocytic necrosis and increased infiltration of megakaryocytes (yellow arrows), which are pathological lesion caused by acute infection with *Salmonella* (+++) Although mild lymphocytopenia or lymphocytosis was observed in F1D4-treated mice (++), no obvious histopathological changes in the spleen were observed in B7D4-treated mice. For the cecum, there was a slight decrease in the number of goblet cells observed in the intestinal mucosal glands throughout all three groups. However, within the PBS group (++), there was a minor degree of lymphocyte infiltration within the lamina propria and mild structural deformation. Pathological observations indicated that treatment with Mabs F1D4 and B7D4 was protective in vivo, by ameliorating histopathological injury and inflammatory infiltration in the live, spleen and cecum of mice infected with peritoneal *Salmonella*. Overall, these results showed that treatment of challenged mice with Mabs F1D4 and B7D4 decreased the colonization of *Salmonella* in the organs and alleviated the organ damage caused by *Salmonella* infection.

**Table 3:** Scoring criteria for the evaluation of histopathological changes in liver, spleen and cecum architecture.

*Liver*:		*Spleen*:		*cecum*	
**Inflammation**		**Lymphoid necrosis**		**Inflammation**	
0	None	0	No significant necrotic foci	0	None
1	Sparse inflammatory infiltrate	1	Occasional small necrotic foci	1	Sparse inflammatory infiltrate
2	Mild inflammatory infiltrate	2	Scattered necrotic foci	2	Mild inflammatory infiltrate
3	Multiple inflammatory lesions	3	Multiple small necrotic foci	3	Multiple inflammatory lesions
4	Severe inflammatory infiltrate	4	Severe necrotic foci	4	Severe inflammatory infiltrate
**Focal necrotic areas**		**Multinucleated giant cells**		**Recess structure deformation**	
0	No significant necrotic foci	0	None	0	None
1	Occasional small necrotic foci	1	1–5	1	1–3
2	Scattered necrotic foci	2	6–10	2	4–6
3	Multiple small necrotic foci	3	11–15	3	7–9
4	Severe necrotic foci	4	15–20	4	10–12

**Table 4:** Pathological score for changes in liver architecture and in spleen architecture.

Organs	Items	PBS	F1D4	B7D4
***Liver***	Inflammation	2	2	1
	Focal necrotic areas	3	2	1
	Total score	5	4	2
	Stratification	++	+	+
***Spleen***	Lymphoid necrosis	3	2	1
	Multinucleated giant cells	3	3	0
	Total score	6	5	1
	Stratification	+++	++	+
***Cecum***	reduction in glandular goblet cells	1	1	1
	Inflammation	0	0	1
	deformation of crypt structure	0	0	1
	Total score	1	1	3
	Stratification	+	+	++

## 4. Discussion

Globally, *Salmonella* is the primary cause of both typhoidal and non-typhoidal gastroenteritis, representing a significant zoonotic public health concern [25]. The emergence of multidrug-resistant and extensively drug-resistant strains of *Salmonella* has limited the efficacy of antibiotic treatment. Vaccines, antiserum, and Mabs are promising approaches for controlling and preventing diseases associated with *Salmonella*. Currently licensed *Salmonella* vaccines include Ty21a and two typhoid conjugate vaccines, Typbar-TCV and TYPHIBEV of *S*. Typhi. Although live attenuated vaccines provide superior cross-protection, their use is limited due to the risk of virulence restoration in immune-compromised individuals. Therefore, there is a focus on developing novel and alternative antimicrobial therapies that provide high specificity, less toxicity, comprehensive immunity and cross-protective efficacy against a broad range of *Salmonella* serovars [17].

Mabs offer numerous innovative options for addressing the challenges associated with neoplastic, inflammatory, and infectious diseases. However, their potential in treating food-borne pathogenic bacterial infections has been largely overlooked. The lack of development and utilization of Mabs therapies for microbial diseases may be attributed to factors such as the overabundance of antimicrobial drugs, high costs, limited market size, and antigenic variability among microbes. However, the landscape of Mabs therapeutics for microbial diseases is evolving in response to increasing antibiotic multidrug resistance, the emergence of new pathogenic microbes, and the development of Mabs cocktail formulations. As a result, there is cautious optimism that more Mabs targeting microbial diseases will be utilized in clinical settings in the years ahead.

Antibody-mediated protection is traditionally associated with opsonization, complement activation, neutralization of toxins and viruses, and antibody-dependent cellular cytotoxicity [26]. In this study, Mab F1D4, targeting FliK has been validated as a potential broad-spectrum drug against *Salmonella*. FliK is a flagellar hook-length control protein affecting the bacterial-type flagellum connecting the filament to the basal body, has been identified as a potential vaccine candidate through *in-silico* prediction and in vivo experiments in previous studies [27,28]. Antibodies directed against this protein could directly enhance the clearance of *S*. *paratyphi* A by phagocytic cells and confer partial protection against *Salmonella* by reducing colonization and invasion during the initial phases of infection [29]. This mechanism may account for the ability of F1D4 to reduce the adherence of *S*. Typhimurium LT2 to host cell surfaces, inhibiting bacterial motility and mitigate mortality *Salmonella*-infected mice. The other screened vaccine candidate, BcsZ, is a type of cellulase that, is secreted and located outside the cell membrane. It plays an important role in various cellular processes such as cell clumping, biofilm formation, flagella-dependent motility, and efficient pathogen-host interactions [30,31]. We hypothesize that antiserum or antibodies generated against BcsZ could potentially block the active site and inhibit *Salmonella* colonization. Notably, our findings demonstrate that Mab B7D4, targeting BcsZ, did display extensive cross-reactivity (26 of 28 strains of *Salmonella*) and a high RPS of 93.33% in *S*. Typhimurium LT2–challenged mice. Besides, B7D4 signifcantly reduced the adherence of *S*. Typhimurium LT2 to host cell surfaces, however, no inhibition in motility was observed in the bacteriostatic zone experiment. The precise mechanism by which B7D4 reduces mortality caused by *Salmonella* infection requires further exploration.

In the present research, a comprehensive investigation into the sites of action and different mechanisms of the Mabs was not conducted. Therefore in future research endeavors, it would be worthwhile to explore whether those two Mabs target specific regions of their respective immunogens to exert broad-spectrum activity. Our serological analyses have confirmed that F1D4 and B7D4 possess the potential to serve as broad-spectrum drugs against *Salmonella*, as well as other non-*Salmonella* bacteria strains. However, cross-immunological protection must be evaluated in a variety of animal models involving challenge with multi-serotype *Salmonella*. Our research indicates that the therapeutic effects of the Mabs are dependent on dosage, with higher doses yielding superior effects. Therefore, optimizing dosages could enhance immune protection. Additionally, combining the two Mabs or pairing them with antibiotic may enable cocktail therapy and significantly reduce antibiotics concentrations while maintaining their efficacy. This approach could minimize drug resistance to some extent. *Salmonella* species are intracellular bacteria, and antibodies tend to have limited efficacy in killing *Salmonella* that reside inside host cells [32]. Specifically, the expression of flagellar proteins is downregulated by *Salmonella* intracellularly, which may account for the effectiveness of antibody treatments during later stages of *Salmonella* infection. Revising the application of cell-penetrating peptides as antibody-type drugs may be of interest for further study.

## 5. Conclusion

In conclusion, in this study, we have successfully generated two Mabs, F1D4 and B7D4, which specifically target FliK and BcsZ, respectively. The titers and cross-reactivity of the Mabs were functionally characterized using ELISAs. Furthermore, we investigated the in vitro effects of these mAbs on bacterial adherence to host cells as well as motility. We also confirmed the safety profile of the antibody in both cell culture and mouse models while demonstrating its protective efficacy against *S*. Typhimurium LT2. The present study has demonstrated the potential of utilizing Mabs F1D4 and B7D4 for immunoassay or immunotherapy against *Salmonella*.

### 5.1 Notice

We state that some figures in this manuscript about the control group (mice mock-treat with PBS) have been published in our another article (DOI: 10.1016/j.ijmm.2021.151508) (Li et al. 2021) [22], because the same control group (mice mock-treated with PBS) was shared by two challenge experiments at the same batch. Detailly, (1) liver (c), spleen (f) and cecum (i) from mice mock-treated with PBS in Fig 6C are identical with Fig 7C (PBS) of the published article; (2) Bacterial load of Liver (A), Spleen (B), Cecum (C) and Feces (D) in Fig 7 from mice mock-treated with PBS are identical with Fig 7D (PBS) of the published article; (3) Survival of mice mock-treated with PBS in Fig 5 are identical with Fig 7E (PBS) of the published article.

## Supporting information

S1 Fig SDS-PAGE analysis of purified Mabs. (TIF)

S2 Fig Analysis of cross-reactivity between Mabs F1D4 (A) and B7D4 (B) with 15 non-Salmonella bacteria strains and the corresponding protein (FliK or BcsZ, as positive control showed in red). Positive samples with a relative OD450 nm >0 are shown in purple (F1D4) or yellow (B7D4). Samples with a relative OD450 nm <0 are shown in black. Relative OD450 nm = OD450 nm of Mabs– 2.1×OD450 nm of serum from mock-immuned mice with PBS.
(TIF)

S3 Fig Gross pathology analysis of liver and spleen at day 7 after infected with 6.4 × 105 CFU S. Typhimurium LT2 intraperitoneally. Each row represents one tissue sample: liver (first row), spleen (second row). Each column represents an independent experimental group immunized with FliK, BcsZ, PBS, F1D4 and B7D4 respectively, the fourth column represents the negative control (NC) without immunization and Salmonella infection.
(TIF)

S1 Table Sequence of the two immunogens FliK and BcsZ used to prepare the monoclonal antibodies. (XLSX)
