# Supplementary figures and images for: Salmonella typhimurium targeting with monoclonal antibodies prevents infection in mice
Source: PLoS Negl Trop Dis. 2023 Dec 4;17(12):e0011579. doi: 10.1371/journal.pntd.0011579 (PMC10745141; doi:10.1371/journal.pntd.0011579)

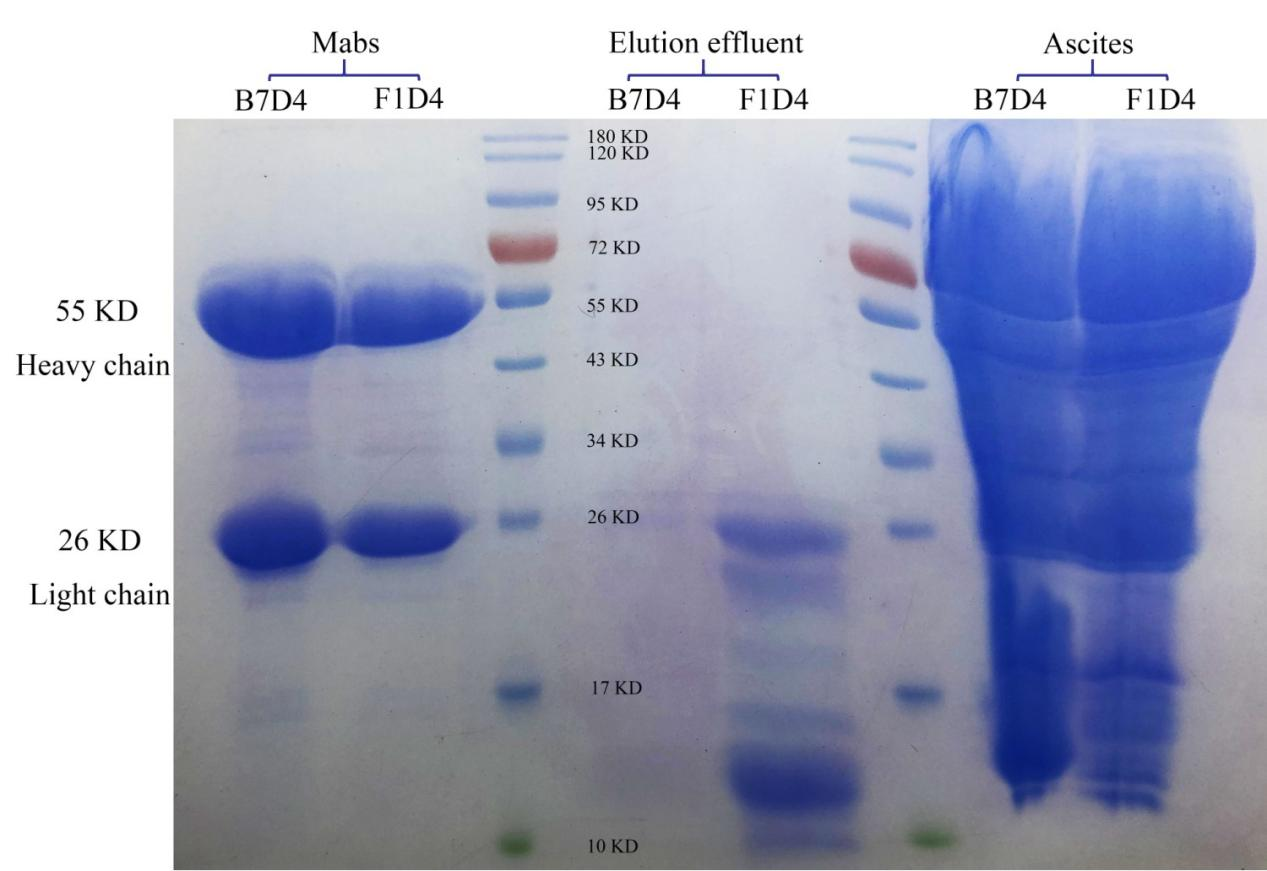

Supplement: S1 Fig — (TIF) [file pntd.0011579.s001.tif]

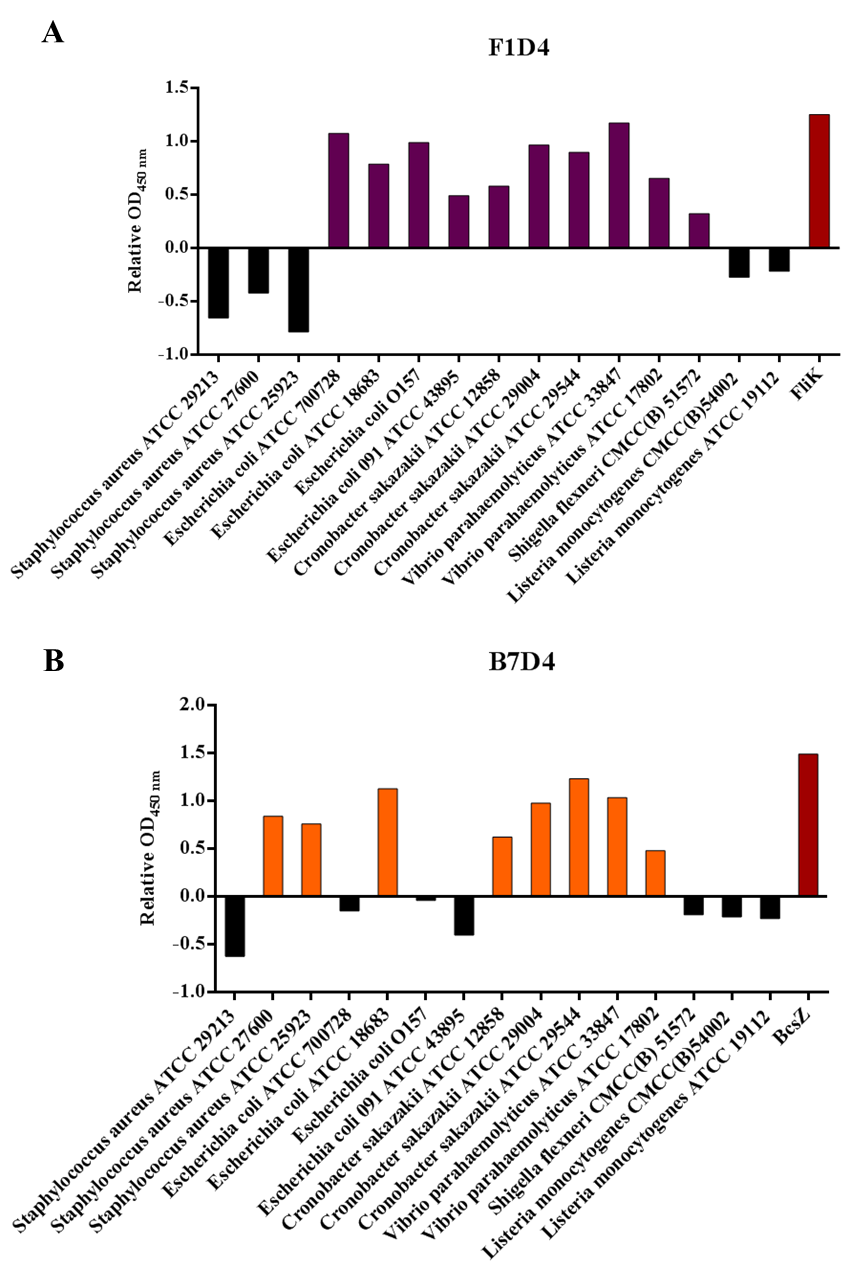

Supplement: S2 Fig — Positive samples with a relative OD450 nm >0 are shown in purple (F1D4) or yellow (B7D4). Samples with a relative OD450 nm <0 are shown in black. Relative OD450 nm = OD450 nm of Mabs– 2.1×OD450 nm of serum from mock-immuned mice with PBS. (TIF) [file pntd.0011579.s002.tif]

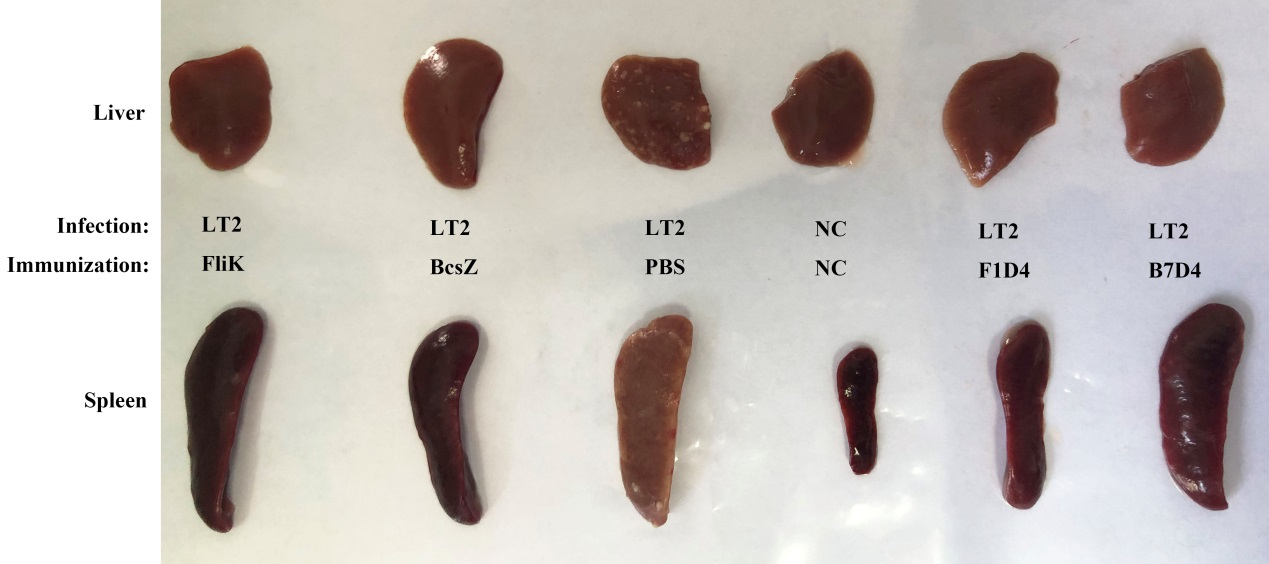

Supplement: S3 Fig — Each row represents one tissue sample: liver (first row), spleen (second row). Each column represents an independent experimental group immunized with FliK, BcsZ, PBS, F1D4 and B7D4 respectively, the fourth column represents the negative control (NC) without immunization and Salmonella infection. (TIF) [file pntd.0011579.s003.tif]
